# STAT3 Regulates MMP3 in Heme-Induced Endothelial Cell Apoptosis

**DOI:** 10.1371/journal.pone.0071366

**Published:** 2013-08-13

**Authors:** Mingli Liu, Nana O. Wilson, Jacqueline M. Hibbert, Jonathan K. Stiles

**Affiliations:** Department of Microbiology, Biochemistry and Immunology, Morehouse School of Medicine, Atlanta, Georgia, United States of America; H.Lee Moffitt Cancer Center & Research Institute, United States of America

## Abstract

**Background:**

We have previously reported that free Heme generated during experimental cerebral malaria (ECM) in mice, is central to the pathogenesis of fatal ECM. Heme-induced up-regulation of STAT3 and CXCL10 promotes whereas up-regulation of HO-1 prevents brain tissue damage in ECM. We have previously demonstrated that Heme is involved in the induction of apoptosis in vascular endothelial cells. In the present study, we further tested the hypothesis that Heme reduces blood-brain barrier integrity during ECM by induction of apoptosis of brain vascular endothelial cells through STAT3 and its target gene matrix metalloproteinase three (MMP3) signaling.

**Methods:**

Genes associated with the JAK/STAT3 signaling pathway induced upon stimulation by Heme treatment, were assessed using real time RT^2^ Profile PCR arrays. A human MMP3 promoter was cloned into a luciferase reporter plasmid, pMMP3, and its activity was examined following exposure to Heme treatment by a luciferase reporter gene assay. In order to determine whether activated nuclear protein STAT3 binds to the MMP3 promoter and regulates MMP3 gene, we conducted a ChIP analysis using Heme-treated and untreated human brain microvascular endothelial cells (HBVEC), and determined mRNA and protein expression levels of MMP3 using qRT-PCR and Western blot. Apoptosis in HBVEC treated with Heme was evaluated by MTT and TUNEL assay.

**Results:**

The results show that (1) Heme activates a variety of JAK/STAT3 downstream pathways in HBVEC. STAT3 targeted genes such as MMP3 and C/EBPb (Apoptosis-related genes), are up regulated in HBVEC treated with Heme. (2) Heme-induced HBVEC apoptosis via activation of STAT3 as well as its downstream signaling molecule MMP3 and upregulation of CXCL10 and HO-1 expressions. (3) Phosphorylated STAT3 binds to the MMP3 promoter in HBVEC cells, STAT3 transcribed MMP3 and induced MMP3 protein expression in HBVEC cells.

**Conclusions:**

Activated STAT3 binds to the MMP3 promoter region and regulates MMP3 in Heme-induced endothelial cell apoptosis.

## Introduction

Adhesion of *falciparum* parasitized erythrocytes to cerebral microvascular endothelium is a major feature of cerebral malaria (CM) pathogenesis that enables infecting parasites avoid splenic clearance [1] by sequestering parasitized red blood cells (pRBC or iRBC) in the brain to cause focal petechial hemorrhages commonly seen in postmortem brain tissues. The surface receptors on vascular endothelial cells such as intercellular adhesion molecule 1 (ICAM-1) and CD36 [2], [3], [4] are responsible for initiating adhesion between iRBC’s and vascular endothelium. During*P. falciparum* malaria infection, the interactions between pRBCs (abnormal erythrocytes) and vascular endothelium induce deleterious endothelial cell responses [5], including inflammation, endothelial activation, and apoptosis that results in the disruption of the blood-brain barrier (BBB) [6]. Apoptosis sequentially occurs in vascular endothelial cells, followed by neuronal and glia cells [7]. pRBC adhesion to the vascular endothelium up-regulate several TNF-superfamily genes and apoptosis-related genes such as Bad, Bax, caspase-3, SARP2, DFF45/ICAD, IFN-g receptor 2, Bcl-w, Bik, and iNOS [8]. In addition, pRBCs increase the expression of ICAM-1 and CD36 [2], [3], [4] which strengthens sequestration, probably through NF-kappa B [2], [3], [4] and MAP Kinase activation [9] and contributes to the pathogenesis of CM.

Increased level of circulating free Heme produced during malaria infection induces inflammation that damages host vascular endothelium and exacerbates fatal CM pathogenesis [10], [11], [12], [13]. Hemeoxygenase (HO) is the rate-limiting enzyme in the degradation of Heme groups to biliverdin, carbon monoxide (CO) and iron. HO-1 protects against cellular stress including oxidative stress, heavy metal toxicity, UV radiation, and inflammation, and prevents deleterious effects of Heme as well as mediating anti-inflammatory and anti-apoptotic functions [14], [15]. HO-1 induction by reactive oxygen species (ROS) and nitric oxide (NO) is involved in regulation of angiogenesis [16], [17] which is necessary to facilitate the repair of injured tissues through inhibition of infiltrating inflammatory cells [18]. It is interesting to note that residual levels of free Heme resulting from a hemoglobinopathy such as sickle cell trait in humans and the hemizygous sickle mice [19] or asymptomatic parasitemia [20] may be protective against severe forms of malaria such as CM [19]. Therefore, there appears to be a minimum threshold level at which free Heme is protective against severe malaria and a level beyond which it is deleterious to the host. This finding suggests that the level of free Heme in circulation during CM pathogenesis or other hemolytic infectious diseases is critical to the extent of tissue damage and should be evaluated and controlled as a strategy for preventing, treating or managing CM and other hemolytic diseases.

Signal transducer and activator of transcription (STAT3) is a signaling cascade activated by pro-inflammatory stimuli and cellular stresses. This protein is located in the cytoplasm in its inactive form and is activated via phosphorylation (pSTAT3) by the Janus tyrosine kinases (JAKs). The active form of STAT3 quickly translocates to the nucleus. pSTAT3 is a potent negative modulator of the Th1-mediated inflammatory response, and also an activator of a variety of genes, which are important for immune modulation [21], [22]. We have previously reported [23] that free Heme generated during ECM model, *P. berghei* (PbA) infection in C57BL/6 mice, is central to the pathogenesis of fatal ECM. Heme induces up-regulation of STAT3 and CXCL10- promotes brain tissue damage in ECM, whereas up-regulation of HO-1 prevents damage. Heme, a by-product of hemoglobin oxidation, has been implicated in the induction of apoptotic death of mouse endothelial and primary HBVEC in a dose- and time-dependent manner [24]. However, the detailed mechanism by which Heme compromises the BBB and the possible functional role of STAT3 during this process remains unknown. In the present study, we further investigate whether Heme induces apoptosis of brain endothelial cells through the STAT3 signaling pathway and identify the target genes for STAT3.

## Results

Elevated hemolysis, indicated by increased level of indirect bilirubin and free Heme plasma concentrations, is a major feature of CM which is linked to disruption and increased permeability of the BBB [25]. In our previous report, we showed that STAT3 activation was crucial to Heme-induced CM pathogenesis [23]. Treatment of mouse brain vascular endothelial cells (MBVEC) with increasing concentrations of Heme, upregulated CXCL10 and HO-1 through STAT3 phosphorylation at Y705. CXCL10 and HO-1 mutually regulate each other [23]. In the current study, we test the hypothesis that the pathophysiological changes in CM caused by high levels of Heme were due to cellular injury to the brain endothelium through activation of STAT3 and its downstream signaling pathways in HBVEC.

### Analysis of Heme-induced JAK/STAT Signaling Pathway using Real time RT^2^ Profile PCR Arrays

**Figure 1:**
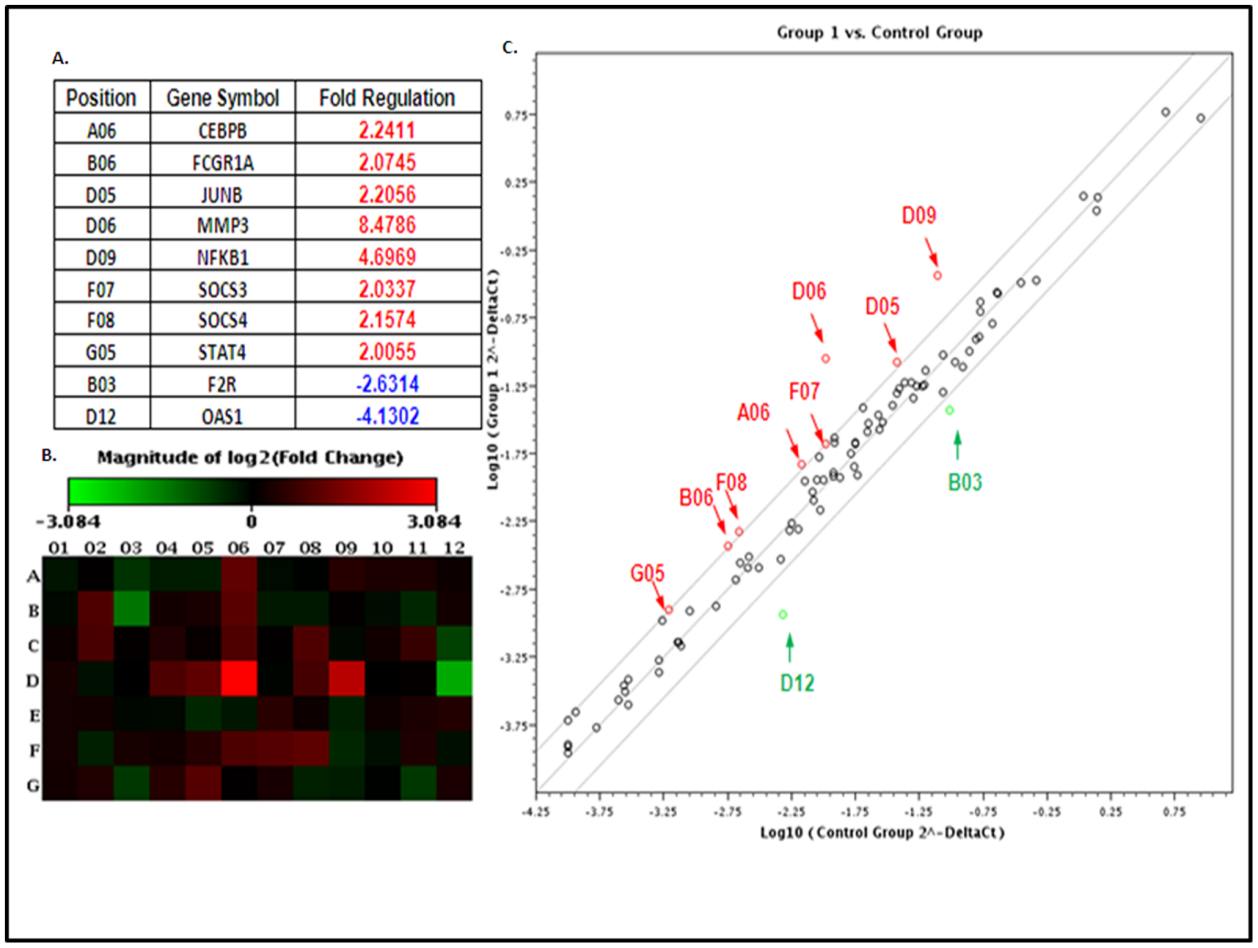
Human JAK/STAT signaling pathway is activated by Heme. Total RNA was extracted and cDNA was synthesized and then used to screen the human JAK/STAT signaling pathway PCR array (SABiosciences, PAHS-039A). Fold changes and p values were calculated using Student’s t-test. A p value<0.05 and a fold change greater than 2.0 were considered to be a significant dysregulation. Figure 1A is a list of up regulated and down regulated genes with fold-change greater than 2. The heat-map (Figure 1B) and scatter plot (Figure 1C) generated are also shown.

Target genes of the JAK/STAT3 signaling pathway induced upon Heme treatment were assessed using real time RT^2^ Profile PCR arrays (SABioscience, PAHS-039A). To avoid the effects of Heme and other factors in serum, we starved the cells with serum-free medium before treatment of Heme to maximize the effects of Heme. HBVEC were serum-starved for 24 h followed by treatment with 30 µM Heme or with vehicle (as control). Total RNA was extracted and subjected to cDNA synthesis and RT^2^ profile PCR array assay. Figure 1A shows a list of up regulated and down regulated genes with fold-change greater than 2 (a P<0.05 was considered to be significantly different). The up regulated genes included STAT-induced gene matrix metallopeptidase 3 (MMP3), apoptosis-related gene CCAAT/enhancer binding protein (C/EBPb), Fc fragment of IgG, high affinity Ia, receptor (FCGR1A), Jun B proto-oncogene (JUNB), nuclear factor of kappa light polypeptide gene enhancer in B-cells 1 (NFκB), suppressor of cytokine signaling (SOCS3), SOCS4 and STAT4. The down regulated genes consisted of coagulation factor II receptor (F2R) and 2′-5′-oligoadenylatesynthetase 1 (OAS1). The heat-map and scatter plot are shown in Figure 1B and 1C.

### Heme Phosphorylates STAT3 and Upregulates MMP3 Protein Levels

**Figure 2:**
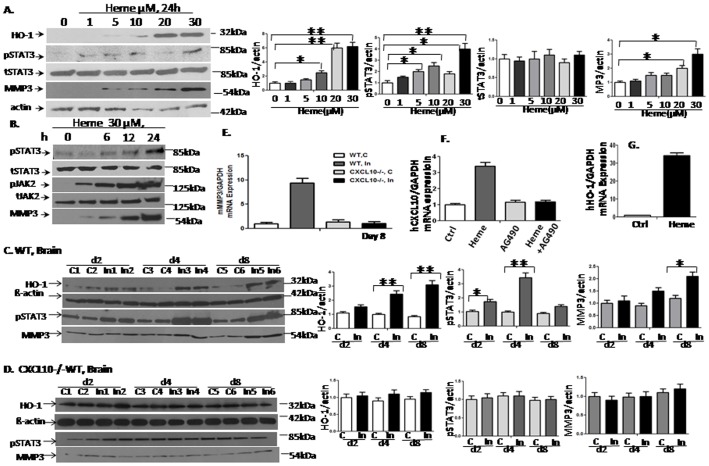
Heme phophorylates STAT3 and upregulates MMP3 protein levels. The effects of Heme on HBVEC as well as activated signaling pathways in HBVEC were examined. HBVEC was treated with different concentrations of Heme as indicated for 24 h. STAT3 activation achieved the maximum effects in HBVEC when treated with Heme with 30 µM (Figure 2A). We performed a time course analysis of Heme treatment to identify when peak STAT3, JAK2 (Tyr1007/1008) activation and endogenous MMP3 induction occurs by Western blot. We found that STAT3 activation peaked at 24 hours. Both of JAK2 (Tyr1007/1008) activation and endogenous MMP3 induction exhibit comparable kinetics in response to Heme (Figure 2B). MMP3 protein was induced by Heme with a similar pattern as HO-1, which appears to be induced later than pSTAT3 (Figure 2A). MMP3 mRNA (Figure 2E) and protein levels (Figure 2C) were up regulated after C57BL/6 mice were infected with *P. berghei* ANKA- PbA (WT, In) at day 8 compared to non-infected controls (WT,C), with a similar trend as observed for STAT3 activation. Interestingly, PbA infection did not up-regulate MMP3 protein in CXCL10-deficient mice, where STAT3 is not activated (Figure 2D). Heme treatment also induced expression of CXCL10 and HO-1 (Figure 2F, G). Corresponding densitometric analyses of the bands performed with the ImageQuant program were shown on the right sides for each panel of Western blot result.

To validate the observations that STAT3 is activated by Heme in HBVEC obtained from RT^2^ profile PCR array, we examined whether Heme can activate STAT3 downstream signaling pathways in HBVEC (Figure 2). After deprivation of serum for one day, HBVEC were treated with different doses of Heme for another 24 h. The dose response of Heme as shown in Figure 2A indicated that STAT3 activation is at 30 µM of Heme. As expected, STAT3 activation indicated by STAT3 phosphorylation (pSTAT3) was evidenced in HBVEC when treated with 30 µM of Heme (Figure 2A). Therefore we utilized this concentration to treat the cells afterwards. In addition, MMP3 protein was induced by Heme with a pattern similar to that observed for HO-1 which appeared later than pSTAT3 (Figure 2A). We also performed a time course of Heme treatment to identify the time point at which peak STAT3 activation occurred as show in Figure 2B. We found that the earliest time point of Heme-induced STAT3 phosphorylation was 6 hours whereas 24 hour is the time point at which peak STAT3 phosphorytion occurred. Subsequently, we performed experiments with Heme treatment using 24 hours time point thereafter. We performed the same time course on JAK2 (Tyr1007/1008) activation by Western blot and endogenous MMP3 induction. Both of them exhibit comparable kinetics in response to Heme (Fig. 2B). This supports our hypothesis that Heme may activate JAK2-STAT3-MMP3 pathway to induce endothelial cell apoptosis. To test whether MMP3 expression is induced *in vivo*, we assessed MMP3 mRNA and protein expression in brain of mice with ECM using the same batch of animal samples, which were collected during the previous project [23]. We found that MMP3 mRNA (Figure 2E) and protein levels (Figure 2C) were up regulated after C57BL/6 mice were infected with *P. berghei*, PbA (WT, In) at day 8 compared to non-infected controls (WT,C), with a similar trend as STAT3 activation [23]. Interestingly, PbA infection failed to up-regulate MMP3 protein in CXCL10-deficient mice, where STAT3 is not activated (Figure 2D). These results suggest that STAT3 functions through its down-stream target gene MMP3 in the pathogenesis of CM. Heme treatment also induced expression of CXCL10 and HO-1 (Figure 2F, G) in human vascular endothelial cells, which have also been observed in mouse endothelial cells [23]. JAK inhibitor AG490 blocked the CXCL10 protein expression caused by Heme thus supporting the observation that Heme-induced CXCL10 upregulation is mediated by STAT3 in MBVEC [23], and that the interactions among Heme-STAT3-HO-1-CXCL10 also exist in HBVEC.

### Heme Induces MMP3 Promoter Activity by Activating STAT3 in HBVEC

**Figure 3:**
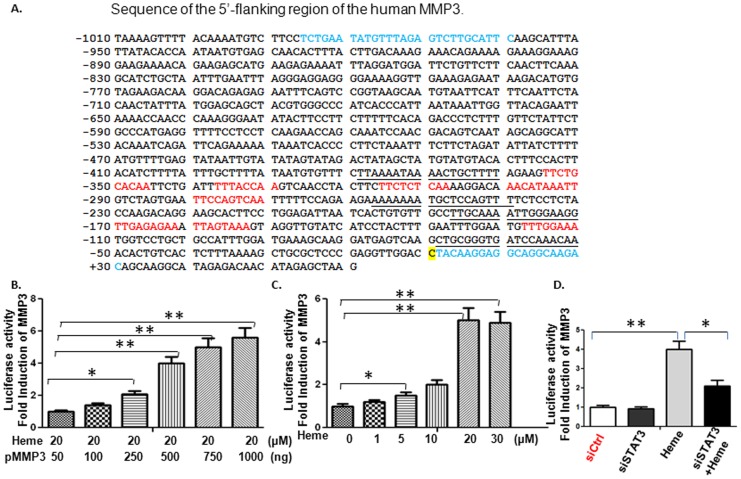
Heme induces MMP3 promoter activity in HBVEC cells. A, Sequence of the 5′-flanking region of the human MMP3 promoter. The primers used to generate MMP3 promoter fragment are shown in blue. The putative STAT3 binding sites, TT(N4–6)AA, are shown in red. Underlined sequences are the primers used to amplify the putative human STAT3 binding sites in MMP3 promoter region in ChIP assay. The “C” shaded in yellow denotes the transcription start site (TSS). To further delineate the transcriptional activity of MMP3 affected by STAT3, HBVEC cells were co-transfected with the MMP3 construct and STAT3-siRNA (siSTAT3) and incubated with Heme. The MMP3 promoter activity was proportional to amounts of MMP3 luc within the 50ng to 1000ng range when treated with Heme (Figure 3B). Figure 3C showed that Heme enhances the MMP3 promoter activity in a dose-dependent manner within a 5 µM to 30 µM range. As shown in Figure 3D, co-transfection with siSTAT3 significantly reduced the Heme-induced luciferase activity of MMP3.

Phosphorylated STAT3 usually binds to the γ-interferon activation sequence (GAS)-like element in the promoter region of targeted genes [26]. Sequence analysis revealed that the MMP3 promoter harbors GAS-like elements TT(N4–6)AA (http://www.cbrc.jp/research/db/TFSEARCH.html), we therefore determined whether STAT3 binds to the MMP3 promoter and how STAT3 transcribes MMP3 gene and induces MMP protein expression in HBVEC cells. To this end, we cloned a human MMP3 promoter (956 bp) into a luciferase reporter plasmid, which was called pMMP3 or MMP3 luc. The location of the 5′ region of the MMP3 promoter construct is indicated in Figure 3A, and the primers used to generate it are shown in blue and described in Methods. The construct was transfected into HBVEC cells, and the activity was assessed after incubation with Heme as indicated in Figure 3. The MMP3 promoter activity was proportional to the amounts of MMP3 luc within the 50 ng to 1000 ng range when treated with Heme (Figure 3B). Figure 3C showed that Heme enhances the MMP3 promoter activity in a dose-dependent manner within a range from 1 µM to 30 µM. To determine if the expression levels of STAT3 would have any effect on the transcriptional activity of MMP3, HBVEC cells were cotransfected with a MMP3 luciferase reporter construct, a siRNA of STAT3 (siSTAT3) and a control siRNA respectively, and then incubated with Heme as indicated. The protein samples were lysed and assayed for luciferase activity. As shown in Figure 3D, siSTAT3 down regulated Heme-induced MMP3 luciferase activity by approximately 47%.

### Tyrosine-phosphorylated STAT3 Binds the MMP3 Promoters in HBVEC Cells

**Figure 4:**
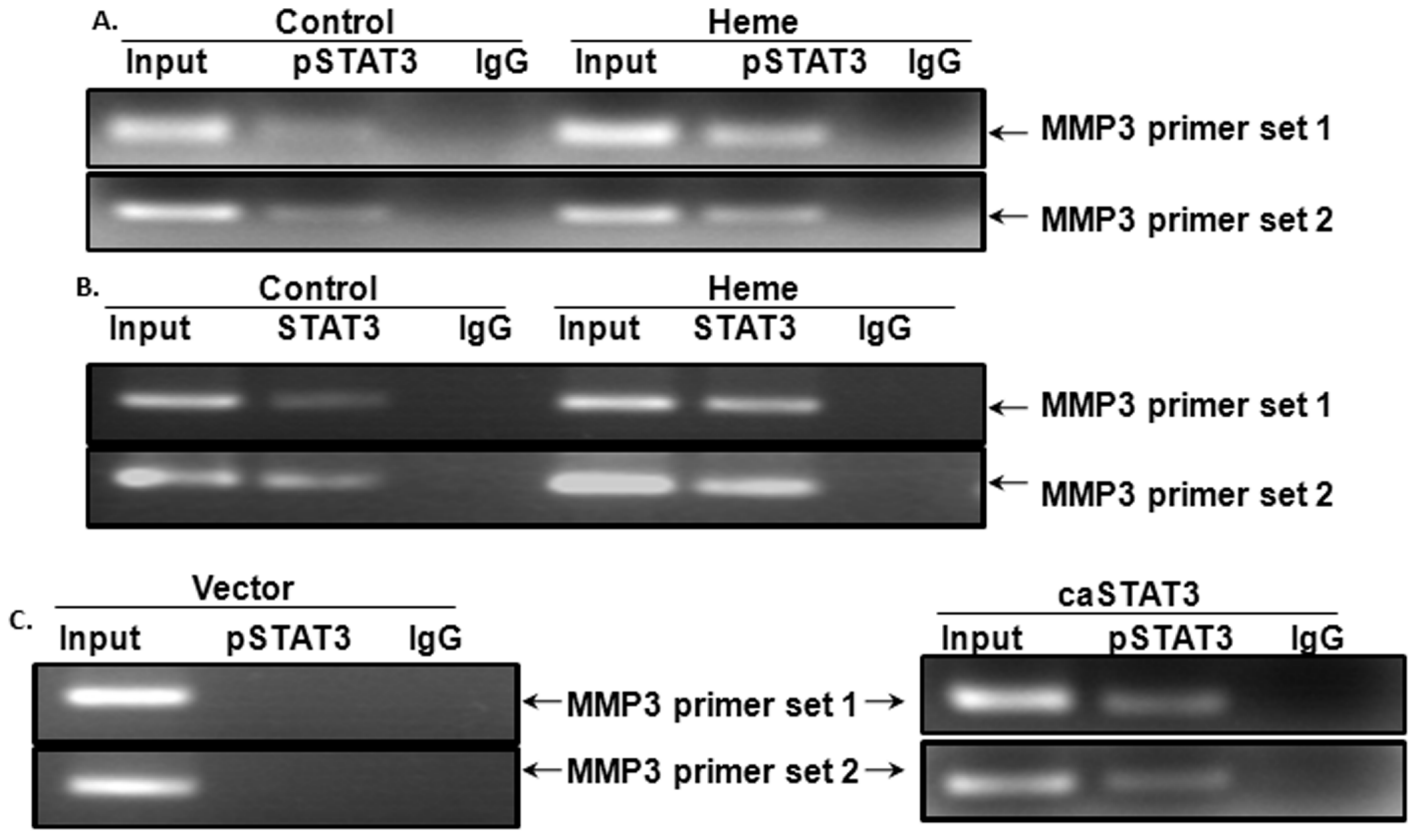
Tyrosine-phosphorylated STAT3 binds the MMP3 promoters in HBVEC cells. In order to determine whether STAT3 is the nuclear protein that binds to the MMP3 promoter, we conducted a ChIP analysis in Heme-treated and -untreated HBVEC cells. As shown in Figure 4A, anti-phopho-STAT3 (Tyr705) antibodies immunoprecipitated MMP3 promoter. A stronger signal was obtained from chromatin of Heme-stimulated HBVEC cells. An anti-STAT3 antibody also immunoprecipitated MMP3 promoter, more strong similar signal was observed from chromatin of Heme-treated compared to -untreated HBVEC cells (Figure 4B). To further validate these findings, we performed a ChIP analysis using HBVEC cells in which STAT3 is constitutively active. We transfected HBVEC with a plasmid constitutively expressing active STAT3 and an empty vectoras control and conducted another ChIP. We found that pSTAT3 co-immunoprecipitated more promoters fragments of MMP3 as shown in the right panel of Figure 4 than the control did (left panel of Figure 4).

When HBVEC cells are treated with Heme, STAT3 is phosphorylated on tyrosine 705 residues, translocated to the nucleus and subsequently activates the transcription of a variety of its target genes [23]. In order to determine whether activated nuclear protein STAT3 binds to the MMP3 promoter, we conducted a ChIP analysis using Heme-treated and untreated HBVEC cells. We generated two specific primer sets for ChIP-PCR analysis. Both sets were designed to amplify promoter regions containing STAT3 putative binding sites, amplifying a region harboring GAS-like elements (Figure 3A). As shown in Figure 4A, anti-phopho-STAT3 (Tyr705) antibodies immunoprecipitated MMP3 promoter. A much stronger signal was obtained from chromatin of Heme-stimulated HBVEC cells than control IgG. As expected, anti-STAT3 antibodies also immunoprecipitated the MMP3 promoter, more strong signals were observed from the chromatin of Heme-treated compared to untreated HBVEC cells (Figure 4B). To further confirm these findings, we performed a ChIP analysis using HBVEC cells in which STAT3 is constitutively active. To this end, we transfected HBVEC with a plasmid constitutively expressing active STAT3, followed by a ChIP assay. We found that pSTAT3 co-immunoprecipitated more fragments of the promoters of MMP3 (right panel of Figure 4) than the empty vector did (left panel of Figure 4). As shown in Figure 4C, amplification was detected with both primer sets designed to amplify different regions having the STAT3 binding sites in MMP3 promoter.

These results, together with the data showing that Heme induced STAT3 phosphorylation and upregulated MMP3 protein levels in HBVEC suggest that STAT3 binds to the MMP3 promoter region and activates MMP3 when stimulated by Heme in HBVEC cells.

### STAT3 Transcribes MMP3 and Induces MMP3 Protein Production in HBVEC Cells

**Figure 5:**
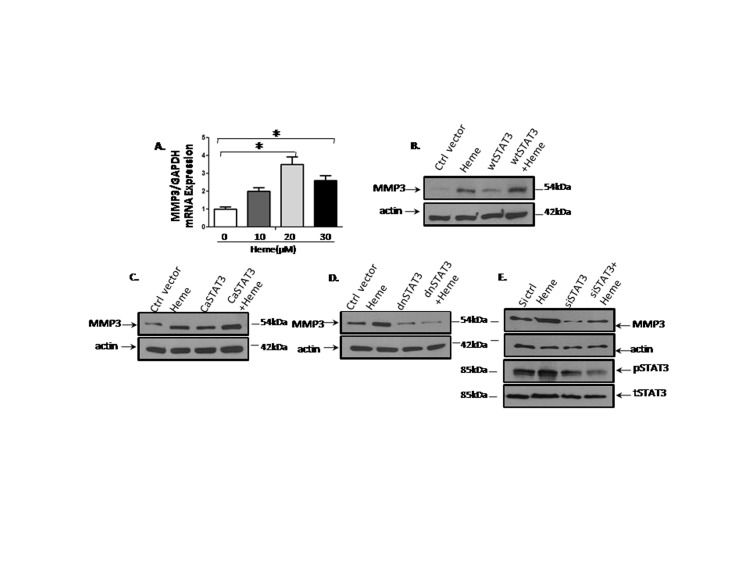
STAT3 transcribes MMP3 and induces MMP3 protein production in HBVEC cells. STAT3 activation of MMP3 has not been previously reported. Therefore, we sought to detect whether STAT3 transcribes MMP3. We stimulated HBVEC cells with Heme, and determined mRNA and protein levels of MMP3 by using qRT-PCR and Western blot. Consistent with the observation that Heme upregulated protein levels of MMP3 as shown in Figure 2A, Heme upregulated mRNA levels of MMP3 in Figure 5A. We then determined whether STAT3 regulates MMP3 using a STAT3-inducible model in HBVEC cells. To this end, HBVEC was transfected with 1 µg of constitutively active STAT3 (caSTAT3), dominant negative STAT3 (dnSTAT3), wild type STAT3 (wtSTAT3) as well as control vector for 24 h. Protein lysates were then made to probe with anti-MMP3 antibody. The results indicate that wtSTAT3 (Figure 5B) and caSTAT3 increased MMP3 expression (Figure 5C) whereas dnSTAT3 reduced MMP3 expression (Figure 5D). When pSTAT3 is reduced by siSTAT3, MMP3 protein expression was inhibited (Figure 5E).

We determined whether STAT3 transcribes MMP3 gene. We stimulated HBVEC cells with Heme, and determined mRNA and protein levels of MMP3 using qRT-PCR and Western blot. Consistent with the observation that Heme upregulated protein levels of MMP3 as shown in Figure 2A, Heme upregulated mRNA levels of MMP3 (Figure 5A). To determine whether STAT3 regulates MMP3, HBVEC were transfected with 1 µg of constitutively active STAT3 (caSTAT3), dominant negative STAT3 (dnSTAT3), wild type STAT3 (wtSTAT3) as well as control vector for 24 h as described previously [27]. Protein lysates were probed with anti-MMP3 antibody. The results indicate that wtSTAT3 (Figure 5B) and caSTAT3 increased MMP3 expression (Figure 5C) whereas dnSTAT3 reduced MMP3 expression (Figure 5D). When pSTAT3 is reduced by siSTAT3, MMP3 protein expression was correspondingly inhibited (Figure 5E).

**Figure 6:**
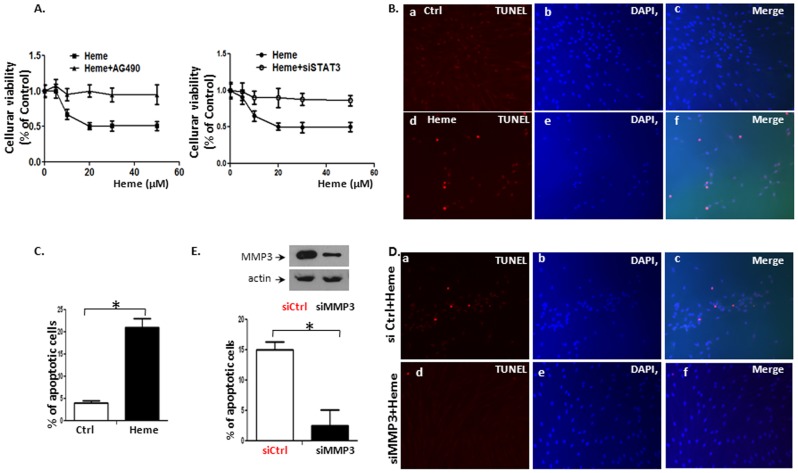
STAT3 induces apoptosis through MMP3 in HBVEC cells. HBVEC were treated with 30 µM of Heme for 24 h followed by evaluation of cell death and apoptosis using MTT and TUNEL assay. Cell death progression in HBVEC cultured were conducted by treating HBVEC cells with AG490 (50 µmol/L) or siSTAT3 as well as corresponding controls followed by incubation with Heme for 24 h, then assayed by MTT measurement. The curves correspond to 3 experiments run in parallel. Heme induced 20%–50% of cell death when treated with 10 to 40 µM of Heme for 24 h (Figure 6A) with 20–30 µM causing maximum effects. The cell death can be rescued by JAK inhibitor AG490 (Figure 6A, left panel) and siSTAT3 (Figure 6A, right panel, *p<0.05, n = 3 triplicate experiments). The reduced cell viability by Heme was further discovered to be caused by cell apoptosis (Figure 6B and 6C). The apoptotic cells were found to be increased by Heme treatment (compare Figure 6B–d vs. 6B–a, 6B–f vs. 6B–c and 6C) using TUNEL assay. When HBVEC cells were transfected with siMMP3 followed by treatment of Heme for 24 h, number of apoptotic cells were reduced (compare Figure 6D–d vs. 6D–a, 6D–f vs. 6D–c and 6E). Upper panel of panel E confirmed specific MMP3 down regulation by siMMP3 by Western blot.

If Heme can induce the apoptosis in human endothelial cells [24], and injury to brain tissues in ECM (mainly in BBB components) through STAT3 signaling pathway as reported by ourselves [23], STAT3 and its targeting gene MMP3 should have crucial roles during this process. Accordingly, the inhibition of JAK/STAT3 and MMP3 should protect endothelial cells from Heme-induced death. To test our hypothesis, we examined the effects of Heme on HBVEC viability. Using the same procedures as described above, HBVEC were treated with 30 µM of Heme for 24 h followed by evaluation of cell death and apoptosis using MTT and TUNEL assay. Heme induced 20%–50% of cell death when treated with 10 to 40 µM of Heme for 24 h (Figure 6A) with 20–30 µM causing maximum effects. Cell death progression assayed by MTT measurement in cultured HBVEC were then conducted by treating HBVEC cells with AG490 (50 µmol/L) or siSTAT3 as well as corresponding controls followed by incubation with Heme for 24 h. The curves corresponding to 3 experiments run in parallel as shown in Figure 6A. As we observed that the cell death can be largely rescued by JAK inhibitor AG490 (Figure 6A, left panel) and siSTAT3 (Figure 6A, right panel, *P<0.05, n = 3 triplicate experiments). These results suggest that STAT3 activation contributes critically to the loss of endothelial cell viability by Heme. The reduced cell viability due to Heme was caused by cell apoptosis (Figure 6B). We randomly chose 10 fields to count the TUNEL positive cells in slide using a 20× microscope objective. Apoptotic indices (% of TUNEL-positive cells/total cell nuclei×100) were calculated after counting cells under a fluorescence microscope [28], [29]. The apoptotic cells were found to be increased by Heme treatment (compare Figure 6B–d vs. 6B–a, 6B-f vs. 6B–c, and 6C) using TUNEL assay. When HBVEC cells were transfected with siMMP3 followed by treatment of Heme for 24 h, apoptotic cells were largely reduced (compare Figure 6D–d vs. 6D–a, 6D–f vs. 6D–c, and 6E). The upper panel of panel E confirmed specific MMP3 down regulation by siMMP3 by Western blot. This indicated that Heme-induces apoptosis in HBVEC by STAT3 activation through MMP3 downstream signaling pathway.

## Discussion

Elevated hemolysis, indicated by increased level of indirect bilirubin and free plasma Heme concentrations, is a major determinant of fatal CM which is associated with increased permeability and disruption of BBB [25]. Dysfunctional vascular endothelium and breakdown of the BBB are hallmarks of pathogenesis of CM [11], [12]. Vascular endothelial apoptosis and disruption of tight junctions (TJs) of endothelium are two adverse factors responsible for compromising the integrity of BBB. Previously [23], we determined that Heme-STAT3-CXCL10 signaling played a central role in ECM pathogenesis and in brain vascular endothelial cell damage using a novel brain vascular endothelial cell assay system [23]. The system involves MBVEC treated with different doses of Heme for 24 h. When MBVEC were treated with increasing doses of Heme, CXCL10 and HO-1 expression were up-regulated through STAT3 phosphorylation at pY705. CXCL10 and HO-1 were mutually regulated [23]. We concluded in that study that the pathophysiological changes in CM were due to the disruption of brain vascular endothelium, which is an important component of BBB through activation of STAT3 signaling stimulated by Heme.

In this study we addressed how Heme disrupts brain endothelium and determined whether Heme could induce endothelial cell apoptosis and disrupt the endothelial TJs. Regarding the relationship between Heme-STAT3 and TJs, some recent studies have demonstrated the adverse effects of Heme-STAT3 on TJs. For instance, oxidative stress induced by Hb/Heme triggers proteolysis of TJ proteins contributing BBB dysfunction [30], [31]. In addition, STAT3 was considered a major signal transducer by which IL-15 increases apoptosis, decreases the TJ protein expression within cerebral endothelia and affects cellular permeability, endocytosis, and intracellular trafficking at the level of the BBB [32]. However, in endothelial cell apoptosis, the causative role of STAT3 as well as its downstream pathways involved in Heme-induced apoptosis is not known and needs further investigation. In this study, we utilized an *in vitro* system consisting of human brain vascular endothelial cells to facilitate an expanded range of inquiry that can be rapidly explored to test the causative role of STAT3 in Heme-induced apoptosis during malaria infection. Our data showed that (1) Heme activates a variety of JAK/STAT3 downstream pathways in HBVEC. STAT3 target genes such as MMP3 [33], [34] and C/EBPb [35], [36] are apoptosis-related genes, and are up-regulated in HBVEC treated with Heme compared with control cells. MMP3 and C/EBPb expression increased 8.48 and 2.24 times respectively (Figure 1). (2) Heme induces HBVEC cell death and apoptosis by activation of STAT3 as well as its downstream signaling of MMP3 (Figure 6) and up-regulation of both CXCL10 and HO-1 expression (Figure 2). (3) Phosphorylated STAT3 binds the MMP3 promoter in HBVEC cells (Figure 4), STAT3 transcribes MMP3 and induces MMP3 protein expression in HBVEC cells (Figure 5).

Activation of vascular endothelial cells in brain by pRBC is a leading cause of encephalopathy associated with malaria. The sequestration of pRBCs in brain microcirculation in CM is due to the erythrocyte membrane protein 1 (pfEMP-1) expressed on pRBCs which adhere to endothelium through endothelial surface receptors [37], mainly ICAM-1 and CD36. The heterogeneity of the vascular endothelium in various locations in the body, characterized by the difference in expression levels of CD36 or ICAM-1 may play an important role in determining the type and severity of malaria. CD36 is an 88-kDa integral protein found on the surface of not only endothelial cells, but adipocytes, platelets, monocytes, and macrophages. ICAM-1 is a 90–115kDa transmembrane glycoprotein expressed on a variety of cell types including endothelial cells [38]. Other endothelial surface antigens including PECAM-1, hyaluronic acid, chondroitin sulfate A (CSA), thrombospondin (TSP), ανβ3, E-selectin, and P-selectinand vascular cell adhesion molecule-1 (VCAM-1) are also reported to be associated with endothelial activation [39], [40], [41], [42]. In contrast to the conclusion that CD36 is a major determinant in severity of malaria, such as CM, some recent results indicated that increased binding to CD36 by parasites is associated with uncomplicated malaria but not CM because little CD36 is expressed on brain microvasculature [38]. Binding to ICAM-1 by parasites is increased in CM and is associated with CM [38]. Chilongola et al suggested that CD36 deficiency may protect against *falciparum* malarial-induced anemia [43]. The reason for the discrepancy in the role of CD36 in malaria is unclear and further studies are required. CM damages microvascular endothelium due to low levels of circulating endothelial progenitor cells (CD34+/VEGF2+ and CD34+/CD133+) [44]. Although activation of vascular endothelial cells in brain by pRBC is a leading cause of encephalopathy, it is worthy to note that direct contact between pRBC and microvascular endothelial cells (EC) may not be required for triggering apoptosis, because soluble factors released from parasitized erythrocytes may also have apoptotic effects on HBVEC and neuroglia cells [45]. Heme, a by-product of hemoglobin oxidation, induces apoptotic death of mouse vascular endothelial and primary human brain microvascular endothelial cells in a dose- and time-dependent way, partly through caspase 3 activation [24]. Astrocyte-derived glutathione attenuates Heme-induced apoptosis in cerebral microvascular cells [24]. Therefore, activation of endothelial cells in brain by pRBC and other factors released by pRBC including Heme are key events leading to encephalopathy of malaria.

In Fig. 2B, 6 hour is the earliest time point at which Heme-induced STAT3 phosphorylation occurs, whereas 24 hour is the time point at which peak STAT3 phosphorytion occurs. This delayed response strongly suggests that Heme indirectly induces STAT3 phosphorylation. Studies from other groups revealed that Heme interacts with JAK2 and alters its conformation [46]. Additionally, in a mouse model of intracerebral hemorrhage, Heme interacts with TLR4 receptor, activates TLR4-mediated inflammatory injury through the MyD88/TRIF signaling pathways [47]. Interestingly, TLR4-associated JAK2 activation was involved in bladder epithelial cell inflammation [48] and phagocytosis in macrophages [49]. In severe malaria cases, patients show increased surface expression of TLR4 on innate immune cells (monocytes and dendritic cells) [50]. Furthermore, certain TLR4 variants have been shown to predispose certain individuals to severe malaria [51]. Based on our previous studies and those of others, Heme-induced delayed STAT3 phosphorylation indicates that Heme indirectly activates STAT3, supporting the hypothesis that Heme activates STAT3 through Heme-TLR4-JAK2-STAT3-CXCL10 pathway.

JAK/STAT3 pathway is involved in cancer [52], immune response [53], ischemia [54], [55] and cellular stress [55], [56]. STAT3 has dual effects on cell survival, as STAT3 can act in deleterious or beneficial roles in cell survival. This effect of STAT3 seems to be cell-type dependent; it may depend on different types of cells, which are located in different tissues and organs. The activation of JAK/STAT3 has been reported in many pathophysiological conditions, especially in the cardiovascular system. JAK/STAT3 signaling activation has been implicated in the protection of the myocardium associated with ischemic and pharmacological pre- and post-conditioning [57], [58], [59], [60]. In the central nervous system, the JAK/STAT3 pathway regulates and improves spinal astrocyte proliferation [61].

In contrast, the apoptotic effects by STAT3 are generated by a variety of mechanisms. STAT3-related apoptotic effects can be oxidative stress-related. Under conditions of increasing oxidative stress, STAT3 can form sulfenic acid that is a characteristic of redox-sensitive proteins, which has a significant role in reducing cell proliferation and viability in human microvascular endothelial cells and cardiac myocytes [55]. β-catenin and PI3K/AKT, which are persistently activated under the influence of inhibition of JAK2 [56], could be the reason for the protective role against apoptosis by STAT3 inhibitor, through inhibition of the JAK2/STAT3 pathway as observed in bovine endothelial cells (BAEC) and human umbilical vein endothelial cells (HUVEC). The potent endothelial protective effects against apoptosis in BAEC or HUVEC, caused by either growth factor deprivation or cell detachment, can be induced by specific inhibition of the JAK2/STAT3 pathway [56]. This cell proliferative effect by the inhibitor of JAK2/STAT3 was even stronger than that of growth factor-rich media, such as 20% fetal bovine serum (FBS) [56]. The vascular endothelial cell tight junctions are essential to the structure of the BBB, therefore any cellular injury to brain endothelial cells will contribute to brain pathology. In monocultures, lipopolysaccharide (LPS) induces death in microglia, which are the brain’s resident immune cells and are among the first responders to brain injury, but not in endothelial cells [62]. Microglial- mediated cell death also appears to involve JAK/STAT3 and NF-κB [62]. In addition, JAK/STAT signaling has been shown to promote and modulate inflammatory processes [62], [63] leading to human BBB dysfunction in HIV infection [64], the latter dysfunction was mediated through CCR5 receptor which is involved in HIV-1 binding to HBVEC and activating the phosphoinositide-dependent kinase-1 (PDK1) and the serine-threonine protein kinase AKT [64]. Furthermore, some studies have shown that JAK2-STAT3 activation plays a role in post-ischemic brain neuronal damage [54] and liver cell inflammation and apoptosis [65], [66].

The reason that only a small fraction of cells stained positive for TUNEL (Figure 6) although all cells were synchronized by 24 hours of serum deprivation and simultaneous Heme stimulation, is not completely clear. Some other genes might be activated as compensation for Heme treatment and offset the action of Heme-induced apoptosis. For instance, activated SOCS could specifically inhibit the function of JAK/STAT3.

MMP3, or stromelysin-1, is a secretory endopeptidase capable of degrading extracellular matrix (ECM), such as collagens, laminins, fibronectin, osteopontin and proteoglycans [67]. MMP3 can be activated by proteases such as plasmin and can also proteolytically activate other MMPs [33]. MMP3 is involved in the remodeling and turnover of the ECM, therefore playing an important role in angiogenesis, wound healing, embryogenesis and morphogenesis and contributing to pathological processes such as cancers, myocardial infarction, fibrotic disorders, rheumatism and osteoarthritis [68], [69]. MMP3 acts as a transcriptional factor and has a novel role in the development, tissue remodeling, and pathology of arthritic diseases through connective tissue growth factor (*CCN2/CTGF*) regulation [70]. MMP3 is responsible for Oncostatin M-specific apoptosis during mammary gland involution [34] and apoptosis of several types of human liver cells [33]. Oncostatin M signaling has been implicated in superseding IL-6 and leukemia inhibitory factor (LIF) to activate both STAT3 and ERK1/2 pathways in mammary epithelial cells leading to differentiation and apoptotic death of mammary epithelial cells *in vivo* [34]. However, the functional study of MMP3 in endothelial cells is poorly understood. This study is the first to report that STAT3 induces apoptotic death of HBVEC cells induced by Heme through MMP3.

It is worth noting that MMP3 is only one of the apoptosis-related genes tested in our RT-PCR array assay (Figure 1). Some other STAT3-targeting genes related to apoptosis needs to be investigated. For instance, C/EBPb is a member of the C/EBP family of transcription factors. Each of the five C/EBP proteins has unique properties regulating cell-type-specific growth and differentiation [36]. Although C/EBPd is a crucial regulator of pro-apoptotic gene expression during mammary gland involution, the role of C/EBPb in induction of apoptosis, especially in the cell components of BBB remains unclear. We truly believe that the protective effects of JAK/STAT3 inhibition against apoptosis of brain endothelial cells and other cell components of the BBB boundary, and subsequent prevention of BBB disruption, are important and warrant further investigation.

## Methods

### Antibodies and Reagents

Polyclonal antibody STAT3, phospho-STAT3, polyclonal antibody JAK2, and phospho-JAK2 (Tyr1007/1008) were purchased from Cell Signaling Technology. Antibody to β-actin was obtained from Sigma-Aldrich. All secondary antibodies used for Western blot were purchased from Calbiochem (La Jolla, CA). AG490 (a JAK2 inhibitor) was obtained from Calbiochem. STAT3 siRNA, MMP3 siRNA and control siRNAwere purchased from Santa Cruz (Santa Cruz, CA). Hemin (Heme) was purchased from Frontier Scientific (*Logan*, UT). All the STAT3-related plasmids were generously provided by Dr. Jackie Bromberg (Memorial Sloan-Kettering Cancer Center, New York) and were generated from the murine stem cell virus (MSCV) vectors with high transfection efficiency into primary cells. Briefly, Wild type Stat3 (wtSTAT3) was cloned into RcCMV-Neo and tagged at the 3′ end with a FLAG epitope. Stat3Y705-F (dnSTAT3) was generated by PCR and cloned into RcCMV-Neo and tagged at the 3′ end with a FLAG epitope [52], [71]. A constitutively activated form of Stat3 (caSTAT3) was bridged or dimerized by two cysteines instead of phosphotyrosines [72].

### Reporter Plasmid Construction

To assay the promoter activity, the 5′-flanking region of the MMP3 gene was inserted into the firefly luciferase reporter vector, pGL3-Basic (Promega), which contained no eukaryotic promoter or enhancer element as described previously [73]. The strategy for cloning of the fragment of the MMP3 gene promoter into pGL3-basic vector was as follows: PCR was performed using the PCR2.1 Topo cloning plasmid which contains the MMP3 gene promoter fragment as a template and 5′- and 3′-primer pairs (the newly synthesized XhoI and HIDIII sites in the primers are underlined), 5′-ATC GCTCGAGTC TGA ATA TGT TTA GAG TCT TGC ATT C-3′, 5′-ATC GAA GCT TGT CTT GCC TGC CTC CTT GTA-3′. The PCR product was then cloned into pGL3-Basic vector. The correct orientation and sequences of plasmid construct were verified by sequence analysis. The unaltered plasmid, pGL3-Basic, was used as a control, and the plasmid, pGL3-SV40 (Promega) contained the firefly luciferase gene driven by the SV40 promoter as a positive control.

### Cell Culture

Human brain vascular endothelial cells (HBVEC; Biowhittaker, Walkersville, MD) were cultured at 37°C with 5% CO2 in endothelial basal medium-2 (Lonza) supplemented with 5% fetal bovine serum (FBS; ATCC, Manassas, VA), growth factors and other supplements including human recombinant epidermal growth factor (hEGF), hydrocortisone, GA-100 (Gentamicin, Amphotericin-B), human recombinant vascular endothelial growth factor (VEGF), recombinant human fibroblast growth factor-B (hFGF-b), recombinant long R insulin-like growth factor (R3-IGF-1), ascorbic acid, heparin, 100 U/ml of streptomycin, and 100 U/ml of penicillin (Lonza). The cells were harvested and passaged at about 70–90% confluence as described previously [45]. At confluence, HBVECs (2×10^5^ cells/ml) were transferred into 35 mm tissue culture dish containing collagen-coated cover slip and incubated at 37°C in 5% CO2 for 24–48 h for future use.

### MTT Assay

HBVEC cells were seeded at 1×10^4^ cells in 100 µl of medium per well into 96-well plates and serum-starved for 24 h, followed by exposing to Heme at 0, 5, 10, 20, 30 and 50 µM for 24 h. 10 µl of MTT reagent (the ratio of MTT reagent to medium is 1∶10) was added into each well and incubated in the dark at room temperature for 2 to 4 h. Absorbance at 570 nm was measured using 650 nm as reference filter using a CytoFluorTM 2300 plate reader and the software CytoFluorTM 2300 v. 3A1 (Millipore Co, Bedford, MA, USA).

### SiRNA Transfection and Retroviral Infection

Small interfering RNA (siRNA) duplexes of STAT3 and MMP3 were designed and purchased from Santa Cruz. A scrambled siRNA, with no homology to any known sequence was used as control. Serum-starved HBVEC cells were transfected with 100 nM specific siRNA or control using Lipofectamine^TM^reagent (Invitrogen, Carlsbad, CA) in serum free OptiMEM-1 medium (Invitrogen) according to the manufacture’s instruction. After six hours of transfection, HBVEC cells were split into two groups and grown in exposure to Heme or not for another 24 h. All studies were done in triplicates. HBVEC cells were transduced with the different MSCVpuro STAT3vectors respectively as previously described [27].

### Western Blotting

Cells were lysed with lysis buffer (50 mmHEPES, 150 mmNaCl, 1.5 mmMgCl2, 1 mmEGTA, 10% glycerol, 1% Nonidet P-40, 100 mm NaF, 10 mm sodium pyrophosphate, 0.2 mm sodium orthovanadate, 1 mm phenylmethylsulfonyl fluoride, 10 µg/ml aproptinin, and 10 µg/ml leupeptin). Samples were separated by SDS/PAGE, and separated proteins were transferred to nitrocellulose membranes and identified by immunoblotting. Primary antibodies were obtained from commercial sources, these antibodies were diluted at the ratio of 1∶1000 according to manufacture’s instruction, while secondary antibodies included HRP-conjugated anti-rabbit and anti-mouse antibodies were obtained from Calbiochem. Blots were developed with Supersignal Pico or Femto substrate (Pierce). A densitometric analysis of the bands was performed with the ImageQuant program (Bio-Rad).

### Real-time RT-PCR Analysis

Cell pellets were stored in Trizol reagent and homogenized in fresh Trizol. Total RNA was isolated from cells using an RNeasy Mini Kit (Qiagen, Valencia, CA). Total RNA was quantified using the Nanodrop N-1000 by Agilent Biosystems (Santa Clara, CA). cDNA was synthesized from the isolated RNA using iScriptcDNA Synthesis Kit (Bio-Rad Laboratories, Inc). Reverse transcription was performed by using random hexamers at 25°C for 5 minutes, 42°C for 30 minutes, and 85°C for 5 minutes. Quantitative PCR were performed using iQSYBR Green Supermix (Bio-Rad Laboratories, Inc.) in a CFX96 Real-Time PCR System machine (Bio-Rad Laboratories, Inc). The data were analyzed using CFX96 Real-Time PCR System (Bio-Rad Laboratories, Inc). Primer sequences for the human CXCL10 and HO-1 were described as previously [23].

### RT^2^ Profiler PCR Array

Total RNA extraction was performed using the RNeasy Mini Kit as described above. The first-strand cDNA synthesis was performed using a RT^2^ First-Strand cDNA Synthesis kit (Qiagen) and 1000 ng of total RNA. cDNA was processed according to the manufacturer’s protocol. Briefly, the cDNA template was combined with RT^2^ Real-Time SYBR Green Master Mix, and RNAse-free water. A final reaction volume of 25 µL was added to each well of the human JAK/STAT signaling pathway PCR array (SABiosciences, PAHS-039A). Finally, pathway focused on mRNA was amplified following the manufacturer’s protocol. Housekeeping genes as well as reverse transcription and positive controls were included in this format. RT-PCR data analysis were performed as follows: alterations in mRNA transcript levels at 24 h with Heme-treated and without Heme-treated groups were initially analyzed using SABiosciences webportal software (http://www.sabiosciences.com/pcrarraydataanalysis.php). Fold changes and P values were calculated using Student’s t-test. A p value<0.05 with a fold change greater than 2.0 were considered to be a significant dysregulation.

### Luciferase Reporter Gene Assay

HBVEC cells were transfected using lipofectamine 2000 (Invitrogen) with 0.75 µg of MMP3 promoter-luciferase construct together with 100 ng of pRL-TK, a cytomegalovirus-Renilla vector to control transfection efficiency. The amount of total DNA transfected was equalized with the appropriate amounts of control vectors. After transfection at different indicated points, cells were harvested and lysed in reporter lysis buffer (Promega, Madison, WI). Luciferase activity was determined by using the Dual Luciferase Kit (Promega, Sunnyvale, CA) and a luminometer (Turner Design, Sunnyvale, CA) according to the manufacturer’s recommendation. All luciferase results were normalized to Renilla activity from the co-transfected pRL-TK plasmid. The data for luciferase activity were expressed as fold induction with respect to control cells and were the mean ± standard error of triplicate samples.

### Immunoflurescence Microscopy

Cells grown in monolayer cultures were fixed with 4% paraformaldehyde in phosphate-buffered saline (PBS), permeabilized with 0.2% Triton X-100, and blocked with 10% fetal calf serum prior to antibody staining. For TUNEL assay, the *in situ* cell death detection kit (TMR red; Boehringer-Mannheim, Mannheim, Germany) was used. The sections were incubated with the TUNEL reaction solution for 60 min at 37°C in the dark. Cover slips were mounted onto slides with Vectashield mounting medium with DAPI (H-1200; Vector Laboratories Inc). Fluorescent images were collected by using a Zeiss LSM510 confocal microscope, and images were captured with LSM software, version 2.3(Carl Zeiss, Wetzlar, Germany).

### ChIP Assay

The ChIP protocol used in this study was adapted from Guo et al [73] and from the protocol recommended by Upstate Biotechnologies. The cells were grown on the 10-cm plates to 85% confluence. Formaldehyde was added to a final concentration of 1%, and the plates were incubated for 10 min at37°C. The cross-linking reaction was stopped by the addition of100 mM glycine containing protease inhibitors (Complete; RocheApplied Science). Cells were washed in dilution buffer (0.01%SDS, 1% Triton X-100, 1.2 mM EDTA, 16.7 mMTris-HCl,150 mMNaCl, pH 8.0 plus protease inhibitors), resuspended in lysis buffer (1% SDS, 10 mM EDTA, 50 mMTris-HCl, pH 8.0 plus protease inhibitors) and sonicated to shear the DNA into 0.3∼3-kb fragments. Following sonication and centrifugation, sheared chromatin was incubated with anti-STAT3, anti-pSTAT3 or rabbit serum (negative control) overnight at 4°C. Then, protein G beads were added and the chromatin was incubated for 2 hours in rotation. An aliquot ofchromatin that was not incubated with an antibody was used as the input control sample. Antibody-bound protein/DNA complexes were eluted and subjected to PCR analysis. Thep rimer sets used to amplify MMP3 promoter with putative STAT3 binding sites were as follows: set 1, F: 5′-TTAAAATAAAACTGCTTTT-3′ and R: 5′AACTGGAGCATTTTTT-3′, which generated a 141-bp product; set 2, F: 5′-TTGCAAA ATTGGGAAGG-3′, and R: 5′-TTGTTTGGATCACCCGCAG-3, which generated a 137-bp product. PCR products were resolved on 1.8% agarose gels.

### Statistical Analysis

The results obtained in this work were expressed as mean ± SEM of at least 2 independent experiments done in triplicate. Paired t-test or ANOVA tests were performed for data analysis, and significant difference was defined as p<0.05.
